# Environmental Enrichment During Adolescence Acts as a Protective and Therapeutic Tool for Ethanol Binge-Drinking, Anxiety-Like, Novelty Seeking and Compulsive-Like Behaviors in C57BL/6J Mice During Adulthood

**DOI:** 10.3389/fnbeh.2018.00177

**Published:** 2018-08-20

**Authors:** Elisa Rodríguez-Ortega, Leticia de la Fuente, Enedina de Amo, Inmaculada Cubero

**Affiliations:** ^1^Departmento de Psicología, Universidad de Almería, Almería, Spain; ^2^CERNEP, Universidad de Almería, Almería, Spain

**Keywords:** environmental enrichment, intermittent drinking in the dark, ethanol, anxiety, compulsivity, novelty-seeking

## Abstract

Repetitive drug/ethanol (EtOH) binge-like consumption during pre-addictive stages favors a transition to addiction in vulnerable organisms. Experimental evidence points to the therapeutic and preventive effects of environmental enrichment (EE) on drug and EtOH addiction; however, little is known regarding EE modulation of binge-like consumption in non-dependent organisms. Here, we explore the impact of early EE on binge-like EtOH consumption: (1) we test whether early EE exposure prevents binge-like EtOH intake (20% v/v) in adult mice under an intermittent drinking in the dark (iDID) schedule; (2) we evaluate the therapeutic effects of EE housing conditions on binge-like EtOH consumption in adult animals; and (3) we compare novelty-seeking and compulsive-like behaviors, and anxiety-like behavior, as measured by the Hole Board (HB) and Elevated Plus Maze (EPM) tests, respectively, in adult EE/standard environment (SE) animals. Adolescent (postnatal day 28; PND28) mice were randomly allocated to two housing conditions (4 animals/cage): EE or SE. At PND67 all the animals were exposed to a schedule of EtOH binge-like iDID. On PND92 half of the animals in each environmental condition (EE and SE) were randomly allocated to two subgroups in a crossover design, where environmental conditions were kept similar to those previously experienced or switched, finally leading to four experimental conditions: EE-EE, EE-SE, SE-SE, and SE-EE. EtOH binge-like consumption continued until PND140, when EPM and HB tests were finally conducted. The main observations were: (1) EE-reared mice showed lower EtOH binge-like intake than SE-reared mice during adulthood, which supports a protective role for EE. (2) when adult EtOH drinking SE-reared mice were switched to EE conditions, a reduction in EtOH binge-like consumption was observed, suggesting a therapeutic role for EE; however, losing EE during adulthood triggered a progressive increase in EtOH binge-like intake. Moreover, (3) EE-housed adult animals with long-term exposure to EtOH binge-drinking showed lower anxiety-like, compulsive-like, and novelty-seeking behaviors than SE-housed mice, irrespective of the specific housing conditions during adolescence. We discuss the primary impact of EE on anxiety-like neurobehavioral brain systems through which it secondarily modulates EtOH binge-like drinking.

## Introduction

Drug and ethanol (EtOH) addiction has been conceptualized as a chronic disorder that involves elements of impulsivity and compulsivity, yielding a composite addiction cycle that develops over three progressive stages ultimately leading to a pathological addiction state (Koob and Volkow, 2010): (1) a binge-intoxication phase driven by the rewarding properties of the drug; (2) a withdrawal phase; and (3) a preoccupation-anticipation phase that precedes renewed drug intake governed by negative reinforcement, enhanced compulsivity and increased sensitivity in the brain stress system (Koob and Volkow, 2010). Drug and EtOH research has been mainly focused on the later dependent stages of the addiction cycle by employing models of EtOH dependence (Yardley and Ray, 2016) and EtOH relapse (Vengeliene et al., 2014). By contrast, early stages of drug addiction and transition to dependence driven by repetitive binge intake episodes remain far less studied. Frequent and intermittent EtOH binge drinking is a typical pattern of excessive EtOH consumption exhibited during the early stages of addiction (Crabbe et al., 2011), and represents an important risk factor for developing addiction in vulnerable individuals (Crabbe et al., 2011; Thiele and Navarro, 2014). Therefore, it is of current interest to provide novel approaches aimed at controlling continued and repetitive episodes of voluntary EtOH binge drinking, and thus protecting vulnerable individuals from progressing to the point of EtOH dependence (Thiele and Navarro, 2014).

EtOH and drug addiction are complex disorders strongly influenced by environmental factors (Nithianantharajah and Hannan, 2006). In this regard, animal research has provided growing evidence that environmental enrichment (EE), a paradigm consisting of housing conditions that include novelty, social interaction and exercise (Crofton et al., 2015), enhances sensory, cognitive, and motor stimulation, which, in turn, translates into important beneficial effects for a variety of neurobehavioral and pathological processes, including drug addiction (Nithianantharajah and Hannan, 2006). Recent research in preclinical models strongly suggests that exposure to EE conditions during adolescence might protect from transitioning to drug addiction and also might work as a therapeutic tool to reduce ongoing drug intake and drug relapse in adult dependent animals (Stairs and Bardo, 2009; Solinas et al., 2010). Thus, rearing C57BL/6J mice in EE conditions reduced locomotor activity induced by acute morphine treatment after chronic morphine exposure (Xu et al., 2014), attenuated acute morphine-induced hyperlocomotion and morphine-induced behavioral sensitization, and also blocked morphine-induced conditioned place preference (CPP; Xu et al., 2007). Additionally, early EE exposure blunted the rewarding effects of heroin, as measured by a CPP test in C57BL/6J mice (El Rawas et al., 2009), and decreased amphetamine self-administration in a fixed ratio schedule in Sprague-Dawley rats (Green et al., 2002). Moreover, EE-reared Sprague-Dawley rats and C57BL/6J mice showed reduced intravenous cocaine self-administration (Green et al., 2010) and a significant reduction in the rewarding properties of cocaine as measured by a CPP test, less activation in response to repeated administration of cocaine injections, and reduced response to repetitive cocaine challenges (Solinas et al., 2009).

Growing experimental evidence highlights the additional ability of EE exposure during adulthood as a therapeutic tool for modulating drug intake, drug reward and drug-relapse in animals with a previous history of continued drug consumption. Thus, exposure to EE during adulthood significantly reduced responses in cue-induced reinstatement of heroin seeking after continued heroin self-administration in a fixed ratio schedule in Long Evans rats (Galaj et al., 2016). EE also reduced methamphetamine-induced behavioral deficits and the risk of drug-withdrawal triggered by drug-relapse in Wistar rats (Hajheidari et al., 2015) and methamphetamine-, heroin- and nicotine- seeking behaviors as well, as assessed in a fixed schedule ratio in Sprague-Dawley rats (Sikora et al., 2018). In addition, EE exposure during adulthood completely eliminated cocaine-elicited behavioral sensitization and CPP, prevented cocaine-induced reinstatement of CPP in C57BL/6 mice (Solinas et al., 2008), eliminated context-induced cocaine-seeking behavior in C57BL/9 mice (Chauvet et al., 2011), and significantly reduced cocaine cue- and stress-induced cocaine reinstatement in Sprague-Dawley rats (Chauvet et al., 2009). Furthermore, exposure to EE reduced the development of incubation of cocaine craving, eliminated already developed incubation in Sprague-Dawley adult rats (Chauvet et al., 2012), and reduced cocaine-seeking behavior during extinction and cue-elicited cocaine reinstatement in Sprague-Dawley rats (Thiel et al., 2009).

Recent evidence has additionally suggested that EE might also prevent and successfully act as a therapeutic tool to reduce voluntary EtOH consumption and EtOH reward. Thus, early EE decreased EtOH consumption, EtOH preference, and motivation to obtain EtOH in limited-access, free-access, and progressive ratio schedules in alcohol-preferring (P) rats (Deehan et al., 2011). It also reduced EtOH self-administration in a fixed ratio scheduled paradigm in Long Evans rats (Deehan et al., 2007), and EtOH preference in a free-choice schedule as well, along with reducing novelty-induced locomotion in spontaneous hypertensive rats (SHR; de Carvalho et al., 2010). Moreover, EE exposure during adulthood blocked reinstatement of EtOH-induced CPP (Li et al., 2015), significantly reduced EtOH consumption, EtOH preference in a two bottle choice task (2BC), and EtOH-induced CPP acquisition in adult C57BL/6 mice (Bahi, 2017a), and also reduced the development of EtOH-induced behavioral sensitization in Swiss-Webster adult mice (Rueda et al., 2012).

Interestingly, the preventive and therapeutic ability of EE might extend to binge-like EtOH consumption exhibited during early stages of the addiction cycle by non-dependent individuals. A mouse model of binge-like EtOH drinking, “Drinking in the dark” (DID; Rhodes et al., 2005, 2007), which triggers high levels of voluntary EtOH consumption (blood EtOH concentrations, BECs of 80 mg/dl or more) over a short period of time (2–4 h; for review see Thiele and Navarro, 2014), has been extensively employed for studying neurobiological and behavioral mechanisms underlying early stages of the addiction cycle and the transition to EtOH dependence (Cox et al., 2013; Thiele and Navarro, 2014; Carvajal et al., 2015). Importantly, some recent studies have shown that social and EE reduces EtOH preference and binge-like EtOH drinking in adult male C57BL/6 mice as measured by a modified, 2BC, DID task. Thus, C57BL/6 mice living in continuous (24 h) or restricted (3 h) EE conditions consumed less EtOH than control mice over 24 h in a DID-2BC procedure after acute stressful conditions (Marianno et al., 2017), and C57BL/6 mice group-housed for 40 days immediately after weaning showed lower binge-like EtOH drinking in a DID-2BC task than isolated-housed mice during the same developmental period (Lopez et al., 2011). Moreover, providing EE was sufficient to counteract high binge-like EtOH intake triggered by chronic social isolation in C57BL/6J mice (Lopez and Laber, 2015).

Taking into account available experimental evidence suggesting the therapeutic and protective abilities of EE exposure during adolescence on drug, EtOH consumption, and EtOH binge-like intake, and because repetitive binge-like consumption during pre-addictive stages might favor transition to addiction in vulnerable organisms (Thiele and Navarro, 2014), it is important to further explore the beneficial impact of EE during adolescence on binge-like EtOH consumption later, during adulthood. The first objective of this work was to evaluate whether early EE exposure during adolescence modulates binge-like EtOH consumption when adult C57BL/6J mice are exposed long-term to an intermittent EtOH DID (iDID) schedule. The second objective was to evaluate the therapeutic effect of EE housing conditions on a stabilized pattern of binge-like EtOH consumption in non-dependent adult animals. To that end, we used a crossover design to examine whether switching from non-enriched to enriched environmental housing conditions during adulthood reduces a stable pattern of voluntary EtOH binge-like intake in an iDID procedure.

There is consistent experimental evidence in drug research indicating that some premorbid behavioral traits, such as high anxiety (Wand, 2005), enhanced compulsivity (Figee et al., 2016), and novelty-seeking behaviors (Iacono et al., 2008; Montagud-Romero et al., 2014; Arenas et al., 2016), are all risk factors that might significantly increase vulnerability to developing drug and EtOH addiction. Interestingly, a number of studies have found that EE exposure reduces anxiety-like responses, compulsive repetitive behaviors and novelty-seeking behaviors, which might represent a potential neurobehavioral mechanism by which EE has a modulatory role on continued drug/EtOH intake. Thus, EE exposure triggered anxiolytic-like responses as measured by the Elevated Plus Maze (EPM) in mice and rats (Peña et al., 2006, 2009; Sztainberg et al., 2010; Ragu Varman and Rajan, 2015; Bahi, 2017b), the Light/dark-box test in mice (Sztainberg et al., 2010; Ragu Varman and Rajan, 2015), and the Elevated Zero Maze (EZM) test in rats (Nobre, 2016). Furthermore, in SHR rats, EE exposure reduced high exploration of a novel environment in an open field (OF) test indicating a reduction in EE-elicited response to novelty (de Carvalho et al., 2010) and significantly attenuated the development of repetitive motor behaviors (Muehlmann et al., 2012; Bechard and Lewis, 2016; Bechard et al., 2016), a sign of compulsivity-like behavior in drug addiction and other disorders (Figee et al., 2016).

Given the aforementioned behavioral evidence that EE exposure exerts a modulatory impact on compulsive-like behavior (Muehlmann et al., 2012; Bechard and Lewis, 2016), anxiety-like responses (Peña et al., 2006, 2009; Sztainberg et al., 2010; Ragu Varman and Rajan, 2015; Nobre, 2016; Bahi, 2017b), and novelty-seeking behavior (de Carvalho et al., 2010), all of which are risk factors to develop EtOH and drug addiction, the third objective in this study addressed whether animals on enriched housing conditions and exposed to continued EtOH binge-like intake show reduced novelty-seeking and compulsive-like behaviors, and anxiety-like behavior, as measured by the Hole Board (HB) and EPM tests, respectively, compared with animals in standard housing conditions.

## Materials and Methods

### Animals and Housing

Male young C57BL/6J mice (Charles River Laboratories, Spain S.A.) were 3 weeks old on arrival to the laboratory. All the animals were housed in groups of four animals in polycarbonate cages (50 × 15 × 25 cm) with stainless steel wire mesh lids and sawdust covering the floor. Each animal’s tail was marked with a nontoxic marker so individual animals could be followed throughout the study. The room was kept at 21 ± 2°C in a 12:12 h light/dark schedule (lights off from 7 am to 7 pm). Animals had *ad libitum* access to chow and water throughout the study with the exception of specific experimental requirements. This study was carried out in accordance with the recommendations of the Bioethical Animal Care Committee at the University of Almeria, Spain. The protocols were approved by the Consejeria de Agricultura y Pesca, Junta de Andalucia, Spain following the animal care guidelines established by the Spanish Royal Decree 53/2013 for reducing animal pain and discomfort.

#### Housing Conditions

**Figure 1:**
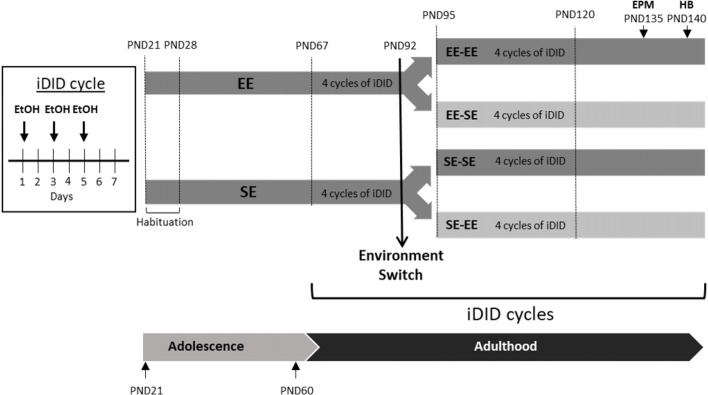
This artwork provides a schematic of the crossover design employed in the study. 1 week after arrival (habituation period from postnatal day 21–28 (PND21 to PND28), animals were randomly allocated to enriched (environmental enrichment, EE) or standard environment (SE) conditions until adulthood (PND67). At PND67, the animals were exposed to four intermittent, Drinking in the Dark (iDID), cycles. A cycle of iDID is composed of three intermittent drinking episodes, each one consisting of 2 h access to EtOH and 3 h in the dark stage. At PND92, half of the animals switched their environmental conditions and half kept their original housing conditions, which resulted in four experimental groups: EE-EE, SE-SE, EE-SE, and SE-EE. From PND95 to PND120, four new cycles of EtOH iDID were administered. Finally, Elevated Plus Maze (EPM) and Hole Board (HB) tests were performed at the end of the study (EPM on PND135; HB on PND140). EPM and HB procedures were performed in ethanol (EtOH)-free days.

After 1 week of acclimation to the laboratory conditions, adolescent postnatal day 28 (PND28) mice were randomly allocated to two possible housing conditions: Environmental enrichment (EE; *n* = 32) or standard environment (SE; *n* = 32; see Figure 1). EE is a paradigm that enhances cognitive, sensorial, and motor stimulation compared to standard housing conditions (for a review see Nithianantharajah and Hannan, 2006). EE housing conditions included a permanent running wheel and a colored plastic object as a hiding place inside the home cage. Additionally, every 5 days a new set of three additional different objects were added to the cages to maintain novelty in the environment. Thus, EE home cages included at least five items (including the running wheel and the plastic hiding place), three of which were changed every 5 days. The objects selected were: PVC tubes, plastic drinking glasses, checkers tiles, ping-pong balls and cotton and carton tubes for nesting purposes. Alternatively, the standard environment included only a single carton tube per cage.

As detailed in Figure 1, half of the animals were kept in EE conditions and half of them in SE conditions from PND28 to PND92. At PND67 (young adults), all of the animals were exposed to EtOH binge-like consumption in an iDID schedule (see below for procedural details). Voluntary EtOH binge-like drinking was recorded for 4 weeks, from PND67 to PND92.

#### Environmental Switch

On PND92, right after the DID session, half of the animals in each environmental condition (EE/SE) were randomly allocated to two subgroups in a crossover design. Thus, environmental conditions were kept as either previously experienced or switched (see Figure 1), finally leading to four experimental conditions: EE-EE, EE-SE, SE-SE, and SE-EE. Exposure to EtOH binge-like consumption in an iDID schedule continued for four additional 7-day cycles, from PND95 to PND120.

### Behavioral Testing

#### iDID

The DID procedure is a mouse model that triggers excessive binge-like EtOH drinking that was originally developed by Rhodes et al. (2005) to generate high levels of voluntary EtOH consumption over a short period of time (2–4 h; Thiele and Navarro, 2014). The iDID is a prolonged and intermittent version of the standard DID (Alcaraz-Iborra et al., 2017). Thus, in the present study, iDID started at PND67 and continued for a total of eight, 7-day cycles, until PND120. In each cycle, on days 1, 3, and 5, 3 h into the dark cycle, animals were transferred to individual cages and had free access to a single bottle of 20% (v/v) EtOH for 2 h. During the DID sessions, animals had free access to regular chow in the cages. On days 2, 4, 6 and 7 mice were kept in their house-cages with *ad libitum* water and food, but no EtOH. EtOH solutions (20% v/v) were freshly prepared every day with absolute EtOH (Panreac Quimica SAU, Barcelona, Spain) diluted in tap water. EtOH bottles and food were weighed before and after each DID session and individual EtOH was measured as g/kg/2 h. An empty cage located in the rack was used for the placement of dummy bottles to measure lost fluid, which was subtracted from total EtOH consumption measures, as a control for fluid spillage.

In order to avoid stress or other unpleasant effects derived from blood extraction procedures to evaluate blood EtOH concentrations (BECs) resulting from binge-EtOH intake, an additional control group provided BECs following 2 h of EtOH consumption in a DID procedure to guarantee that animals reached high BECs in the procedure employed. Hence, an independent cohort of male C57BL/6J mice (*n* = 12; PND81) was trained for EtOH binge-drinking in a standard DID paradigm. On days 1, 2 and 3, animals had a 2-h access to a single bottle of EtOH (20% v/v) and chow. On day 4, a 2-h test session was performed, since there is previous evidence that mice achieve binge levels of EtOH consumption in such a short period of time (Olney et al., 2017). Immediately after the test session, individual tail blood samples (10 μl) were collected from each animal and centrifuged, and 5 μl of plasma from each sample was analyzed for BEC (mg/dl; Analox Instruments, Lunenburg, MA, USA; Carvajal et al., 2015).

From PND135 to PND140, once the animals in the study had completed a total of 24 episodes of binge-EtOH iDID, anxiety-like responses, novelty-seeking and compulsivity-like behaviors were assessed as measured by the EPM and the HB. In order to avoid any confounding withdrawal effect, EtOH binge-like sessions of iDID continued from PND120 to the end of the study on PND140. Nonetheless, EPM and HB procedures were always carried out on EtOH-free days.

#### EPM

The EPM apparatus employed in the present study was made of two open (30 cm × 5 cm) and two enclosed arms (30 cm × 15 cm × 5 cm) with a common central square (5 cm × 5 cm) all made of black Plexiglas (Cibertec, S.A., Madrid, Spain). The apparatus was elevated 50 cm from the floor and had photocells in order to constantly register mouse positions. Indirect halogen illumination provided 120 lux onto the open arms and 35 lux onto the closed ones. Animals were acclimated to the experimental room over 2 days for 2 h. On test day (day 3; PND135), animals were placed individually in the center of the maze always facing an open arm, and their behavior was automatically recorded by photo cells for 5 min (Cibertec Software, S.A., Madrid, Spain). An entry was registered when all four paws of the animal were placed into an arm. After every individual trial, the floor and walls of the apparatus were extensively cleaned with soapy water and totally dried with paper towels. Percentage of total number of entries to open arms and percentage of total time spent in open arms were registered for anxiety-like behavior assessment (Hargreaves and McGregor, 2007; Walf and Frye, 2007; Alcaraz-Iborra et al., 2017; Marianno et al., 2017). The total number of arm entries were recorded to assess locomotor activity (Hargreaves and McGregor, 2007; Alcaraz-Iborra et al., 2017; Marianno et al., 2017).

#### HB

The hole board apparatus was made of Plexiglas (28 × 28 × 20.5; red walls and black floor), and the surface was covered with 16 equidistant holes with 3-cm diameters (Cibertec, S.A., Madrid, Spain). A set of photocells located below the hole surface detected total number of head dipping. Animals were acclimated to the experimental room over 2 days for 2 h. On test day (day 3; PND140), at the beginning of the session, mice were placed in the center of the apparatus and allowed to explore for 10 min. After every individual trial, the floor and walls of the apparatus were extensively cleaned with soapy water and totally dried with paper towels. Total number of head dips performed was automatically registered to evaluate novelty-seeking behavior (Abreu-Villaça et al., 2006; Mateos-García et al., 2015) and total repetitions of head dips was automatically recorded and later analyzed as a measure of compulsive-like behavior (Chao et al., 2010; Moy et al., 2014).

### Data Analysis

Average EtOH consumption data (g/k/2 h) obtained during the DID sessions were collapsed into eight, 7-day cycles. In order to evaluate the impact of environmental conditions on adult EtOH binge-like drinking (from PND67 to PND120; see Figure 1), a repeated measures (4 × 8; environment × cycle) analysis of variance (ANOVA) was performed. When significant interactions emerged, Bonferroni *post hoc* analyses were performed to compare EtOH consumption by experimental groups in each cycle. Additionally, planned paired *t*-tests were performed across key cycles within each group to evaluate consumption over time. Thus, we chose to compare cycle 1 vs. 4, 4 vs. 5, 5 vs. 8 and 4 vs. 8, using different error terms for each set of comparisons, which allowed a panoramic view of EtOH consumption progression across the cycles in each group in response to environmental manipulations. Due to the small number of comparisons made for each independent group, it was not necessary to strictly adjust for familywise error types. Finally, data recorded in the EPM (percentage of entries to open arms, percentage of time in open arms, and total number of arm entries) and HB (number of head-dips and number of head-dip repetitions) were analyzed using an independent one-way ANOVA. Significance was set at *p* < 0.05 and Grubb’s test was used to identify and exclude outlier data in all the analysis. All data in this report are presented as mean ± standard error of the mean (SEM).

## Results

### Effects of Environmental Switch on Steady EtOH iDID Binge-Like Intake

**Figure 2:**
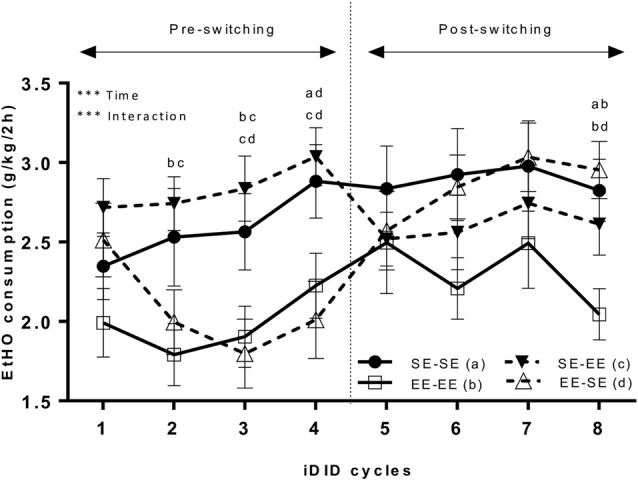
This artwork represents averaged (means ± standard error of the mean; SEM) EtOH (20% v/v) consumption (g/k/2 h) by EE and SE mice during pre-switching and post-switching stages. The vertical dotted line indicates the switch-point of environmental housing conditions. ***p < 0.001, relative to EE EtOH intake in the iDID cycle. *Post hoc* results comparing experimental conditions are represented as a combination of letters.

Figure 2 shows data representing average EtOH (20% v/v) binge-like intake over eight cycles (see Figure 1). Because Mauchly’s Test for the repeated measures ANOVA (4 × 8; environment × cycle) showed the unique non-compliance of the sphericity assumption (Mauchly’s Test: χ^2^ = 0.22, *p* < 0.0001), the Greenhouse-Geisser correction was used to estimate both repeated measures and interaction effects. The results showed a statistically significant cycle main effect (*F*_(4.53,272.02)_ = 6.94, *p* < 0.0001; η^2^ = 0.10) and interaction (environment × cycle; *F*_(13.60,272.02)_ = 3.61, *p* < 0.0001; η^2^ = 0.15). The main factor environment (*F*_(3,60)_ = 2.39, *p* = 0.07) did not attain statistical significance. Bonferroni *post hoc* analyses showed that EE-EE animals significantly consumed less EtOH solution than the SE-SE group in cycle 2 (*p* = 0.02). In cycle 3, SE-EE EtOH binge-like intake was larger than in the EE-SE (*p* = 0.007) and EE-EE (*p* = 0.01) groups. In cycle 4, statistically significant differences in EtOH binge-like intake emerged between the EE-SE and SE-SE groups (*p* = 0.03) and EE-SE and SE-EE groups (*p* = 0.008), thus, the EE-SE group significantly consumed less EtOH than the SE-EE and SE-SE groups. Finally, in cycle 8, EE-EE animals consumed less EtOH than SE-SE (*p* = 0.01) and EE-SE (*p* = 0.004) animals. One mouse in the SE-SE group was excluded as an outlier.

Additional planned *t*-tests within groups allowed us to analyze consumption dynamics over cycles in each experimental group (Table 1). Thus, the EE-EE group showed no statistically significant differences in cycles 1 vs. 4 (*t* = −1.22, *p* = 0.23; *df* = 15), 4 vs. 5 (*t* = −0.83, *p* = 0.42; *df* = 15), 4 vs. 8 (*t* = 0.94, *p* = 0.36; *df* = 15), nor 5 vs. 8 (*t* = 1.92, *p* = 0.07; *df* = 15), indicating that EtOH binge-like intake was stable over time in the EE-EE group. Planned *t*-tests in the EE-SE group showed statistically significant differences in cycles 1 vs. 4 (*t* = 3.16, *p* = 0.006; *df* = 15), cycles 4 vs. 5 (*t* = −3.42, *p* = 0.004; *df* = 15), and 4 vs. 8 (*t* = −3.85, *p* = 0.002; *df* = 15), but not for cycles 5 vs. 8 (*t* = −1.91, *p* = 0.07; *df* = 15). Thus, the EE-SE group showed a progressive reduction in EtOH binge-like intake from cycle 1–4 followed by a dramatic increase when losing EE, which slightly escalated from cycle 4–8. When EtOH intake by the SE-SE group was analyzed across cycles, paired *t*-tests showed significant statistical differences in cycles 1 vs. 4 (*t* = −2.16, *p* = 0.02; *df* = 15), but not in cycles 4 vs. 5 (*t* = 0.31, *p* = 0.75; *df* = 15), cycles 4 vs. 8 (*t* = 0.35, *p* = 0.72; *df* = 15), nor cycles 5 vs. 8 (*t* = 0.06, *p* = 0.94; *df* = 15), indicating that EtOH binge-like consumption by SE-SE mice increased from cycle 1–4, reaching a plateau at this point across the next four cycles. Planned comparisons for the SE-EE group showed significant statistical differences in cycles 4 vs. 5 (*t* = 4.05, *p* = 0.001; *df* = 15), a clear tendency in 1–4 (*t* = −2.01, *p* = 0.06; *df* = 15), 4 vs. 8 (*t* = 1.89, *p* = 0.07; *df* = 15), and 5 vs. 8 (*t* = −0.56, *p* = 0.57; *df* = 15). Therefore, the SE-EE group escalated EtOH consumption from cycles 1–4 and then reduced EtOH binge-like intake right after environmental switching, keeping steady EtOH consumption for the rest of the experiment across cycles (see Table 1).

**Table 1:** Shows planned paired *t*-tests performed on key intermittent drinking in the dark (iDID) cycles within each group to evaluate ethanol (EtOH) binge-like consumption over time. *p < 0.05; **p < 0.01; *df* = 15.

	EE-EE				EE-SE			
Cycles	1 vs. 4	4 vs. 5	4 vs. 8	5 vs. 8	1 vs. 4	4 vs. 5	4 vs. 8	5 vs. 8
*T* test	−1.228	−8.3	0.94	1.92	3.167	−3.427	−3.857	−1.91
*P*	0.238	0.42	0.362	0.074	0.006**	0.004**	0.002**	0.075
	**SE-SE**				**SE-EE**			
Cycles	1 vs. 4	4 vs. 5	4 vs. 8	5 vs. 8	1 vs. 4	4 vs. 5	4 vs. 8	5 vs. 8
*T* test	−2.61	0.319	0.355	0.067	−2.019	4.055	1.898	−0.596
*P*	0.02*	0.754	0.728	0.947	0.062	0.001**	0.077	0.578

A control naïve group housed in the same conditions as the SE group was employed to test BECs resulting from 2 h EtOH exposure in a DID procedure. Animals showed an average of 2.47 g/kg/2 h of EtOH consumption and revealed intoxicant BEC levels higher than 80 mg/dl (average = 92.97 mg/dl), which is the standard criterion to constitute a binge episode (NIAAA National Advisory Council, 2004; data not shown). One mouse was excluded as an outlier.

### Anxiety-Like Responses as Measured by the EPM

**Figure 3:**
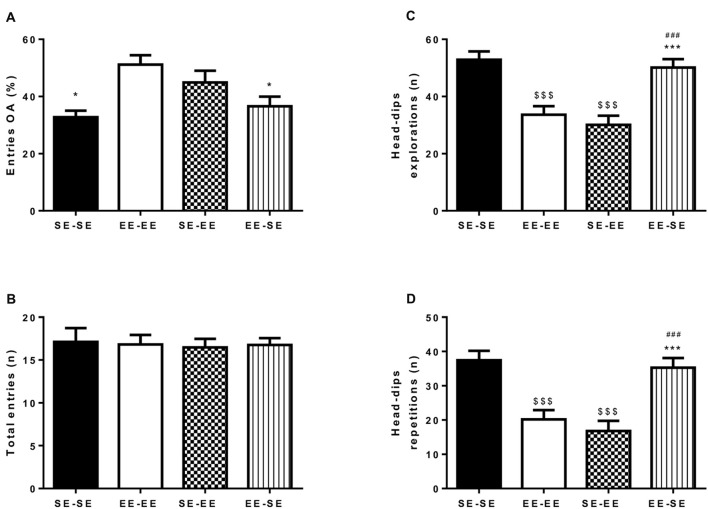
This artwork represents averaged (means ± SEM) percentage of entries to open arm (OA; **A**) and total number of arms entries **(B)** observed in the EPM by all the experimental groups at postnatal day 135 (PND135). Graph **(C)** represents the average number of head-dip explorations; and graph **(D)**, head-dip repetitions observed in the HB test by the experimental groups at PND140. *p < 0.05 and ***p < 0.001 relative to the EE-EE group; ^$$$^*p* < 0.001 relative to SE-SE mice and ^###^*p* < 0.001 relative to SE-EE mice.

Figures 3A,B depicts percentage of entries to open arms (A) and total number of entries to arms (B). An independent one-way ANOVA performed on percentage of entries in open arms showed statistically significant group differences (*F*_(3,52)_ = 4.98, *p* = 0.004; η^2^ = 0.22; Figure 3A). Bonferroni *post hoc* analysis revealed that the EE-EE group showed significantly lower anxiety-like behavior than the SE-SE (*p* = 0.01) and EE-SE (*p* = 0.02) groups. The absence of group differences in total arm entries (*F*_(3,52)_ = 0.04, *p* = 0.98) indicates that all the animals in the different environmental conditions showed similar locomotor activity levels (Figure 3B). In brief, mice housed in EE conditions and drinking continued EtOH in an iDID procedure from PND67 to PND135 showed lower anxiety-like responses as measured by the EPM during adulthood than animals housed in SE conditions in the same period. Furthermore, loss of enrichment in their environment from PND92 was associated with increased anxiety-like behaviors, as exhibited in the EE-SE group compared to the EE-EE group on PND135.

### Novelty-Seeking Responses and Compulsive-Like Behavior as Measured by the HB

Figures 3C,D show total head-dip explorations (C) and total head-dip repetitions (D). Independent one-way ANOVA performed on total number of head-dip exploration data by animals in different housing conditions (Figure 3C) revealed significant group differences (*F*_(3,60)_ = 14.50, *p* < 0.001; η^2^ = 0.42). Additional Bonferroni *post hoc* analysis revealed a significantly higher number of head-dip explorations in the SE-SE group than in the EE-EE (*p* < 0.0001) and the SE-EE (*p* < 0.001) groups on PND140. Furthermore, at this time point, the EE-SE group also showed more head-dip exploration than the EE-EE (*p* = 0.002) and the SE-EE (*p* < 0.0001) groups.

Regarding total head-dip repetitions, an independent one-way ANOVA performed on data showed significant group differences (*F*_(3,60)_ = 13.81, *p* < 0.0001; η^2^ = 0.40; Figure 3D). Bonferroni *post hoc* analysis revealed higher total head-dip repetitions in the SE-SE group than in the EE-EE (*p* < 0.0001) and the SE-EE (*p* < 0.0001) groups on PND140. The EE-SE group had a greater total number of head-dip repetitions than the EE-EE (*p* = 0.002) and the SE-EE (*p* < 0.0001) groups in this time window.

## Discussion

There are four main observations in this study. First, adult mice reared during adolescence in EE housing conditions showed a significantly lower EtOH binge-like intake than adult mice reared in SE housing conditions, indicating a protective role for EE. Moreover, while SE-SE animals showed an escalation in EtOH consumption over the first four cycles of iDID, EE-EE animals maintained a stable and low intake over time. Second, when adult SE-reared mice exposed to EtOH binge-like iDID for a long duration (four 7-day cycles) during adulthood were switched to EE conditions (SE-EE), an immediate reduction in EtOH binge-like consumption was found, suggesting a therapeutic role for EE. Third, when EE-reared mice exposed to a long duration of EtOH bingeing in an iDID paradigm lost EE during adulthood (EE-SE group), a progressive and marked increase in EtOH binge-like intake was observed over time, matching EtOH consumption by the SE-SE group. Fourth, adult animals pre-exposed to eight, 7-day cycles of EtOH binge-drinking and housed under EE conditions at the time of testing, showed lower anxiety, compulsive-like and novelty-seeking behaviors than SE housed mice, irrespective of the specific housing conditions during adolescence.

It is worth noting that evidence in our study pointing to the protective and therapeutic benefits of EE was based upon comparison with control standard housing conditions (SE), which consisted of mice housed in groups of four animals with a carton tube for nesting purposes. Other studies employing isolated animals as EE control condition also showed that early social isolation triggered EtOH consumption in rats and mice (Deehan et al., 2007, 2011; Lopez et al., 2011; Lopez and Laber, 2015). Taking into account that both isolated (previous studies) and SE conditions (present results) correlated with EtOH consumption, one first suggestion from the present data is that socialization itself (SE animals) is a necessary, but not sufficient, condition to moderate binge-like EtOH consumption in adult animals; rather, additional motor, cognitive, sensorial, and visual stimulation, as selected in our present study, seems necessary to achieve a significant reduction in EtOH binge-like consumption. Nonetheless, we cannot rule out that additional methodological differences, such as exposure time to EE conditions, specific EE housing conditions and protocols of exposure to EtOH consumption, as well as the strain, sex, or species of animals employed, all affect behavioral outcomes in EE studies (Crofton et al., 2015) and might explain existing discrepancies among laboratories on the effects of EE on EtOH consumption.

The first important finding in our study is that adult mice reared during adolescence in EE housing conditions and exposed long-term to EtOH binge-like iDID (eight 7-day cycles) during adulthood, showed a lower EtOH binge-like intake than mice reared in SE housing conditions. Thus, *post hoc* Bonferroni tests showed higher EtOH binge-like intake in SE animals than in EE animals in cycles 2, 3 and 4, indicating the ability of EE to control the development of spontaneous EtOH binge-like drinking. Moreover, specific planned comparisons revealed that while SE-SE mice showed a progressive increase in EtOH binge-like consumption over time, from cycle 1–4, reaching a plateau at 4th cycle, EE-EE animals did not escalate and kept a stable EtOH intake over these four cycles, which supports a protective role for EE during adolescence to prevent EtOH binge-intake escalation. Present data extend previous observations from pre-clinical models showing that early access to EE conditions protects the development of drug intake including morphine (Xu et al., 2007, 2014), cocaine (Solinas et al., 2009; Green et al., 2010), heroin (El Rawas et al., 2009), amphetamine (Green et al., 2002), sucrose (Grimm et al., 2008, 2013), and EtOH (Deehan et al., 2007, 2011; de Carvalho et al., 2010; Holgate et al., 2017). Frequent and intermittent EtOH binge drinking is a typical pattern of excessive EtOH consumption for experiencing intoxication, exhibited during early stages of the addiction cycle (Crabbe et al., 2011), that represents a substantial risk factor predicting the development of EtOH addiction (Crabbe et al., 2011; Thiele and Navarro, 2014). Control SE animals in the study tested for BECs after 2 h of EtOH exposure in the DID procedure showed BECs ≥ 80 mg/dl, which is the standard criterion to constitute a binge episode (NIAAA National Advisory Council, 2004). Consequently, present results showing reduced EtOH binge-like drinking in EE- vs. SE-reared animals provide novel data raising the exciting possibility that exposure to EE during adolescence could protect vulnerable organisms from developing excessive EtOH binge-like consumption and subsequently transitioning to EtOH addiction later during adulthood, even under long-term EtOH availability conditions.

The second main observation is centered on the therapeutic impact of EE housing conditions introduced in adulthood, on binge-like EtOH consumption. When adult SE-reared animals drinking EtOH for four iDID cycles were switched to EE (SE-EE group), planned comparisons showed an immediate and marked reduction in EtOH binge-like consumption from cycle 4–5. Moreover, Bonferroni analysis revealed that the pre-switching SE-EE vs. EE-EE significant differences in EtOH binge-like intake in cycle 4, disappeared right after environmental switching until cycle 8, where those differences emerged again. Taken together, these data indicate, first, the therapeutic ability of EE to modulate a stabilized pattern of binge-like EtOH intake in adult organisms and, second, the temporal and limited nature of that modulation. Present results are in agreement with previous reports showing that EE ameliorates EtOH consumption and preference in a 2BC paradigm in mice (Bahi, 2017b) and counteracts high binge-like EtOH intake triggered by chronic social isolation during early development as well (Lopez and Laber, 2015). Also, the present data supports previous studies showing the therapeutic impact of enrichment conditions on drug addiction such as heroin (Galaj et al., 2016), methamphetamine (Hajheidari et al., 2015, 2017), and cocaine (Solinas et al., 2008; Chauvet et al., 2009, 2011, 2012; Thiel et al., 2009). Future studies extending total time of testing are needed to further explore the temporal dynamic of the beneficial therapeutic impact of EE exposure on binge EtOH intake during adulthood.

The third relevant observation is centered on the impact of losing EE on EtOH binge-like drinking (EE-SE group). Planned comparisons conducted on EE-SE data showed that EE-SE mice first reduced spontaneous EtOH intake over the first four cycles of iDID, confirming the beneficial impact of early EE to modulate spontaneous EtOH binge-like consumption during adulthood. Interestingly, when adult EE-reared mice lost EE, a progressive and marked increase in EtOH binge-like intake over time was observed. Thus, losing EE triggered a clear increase in EtOH consumption in the EE-SE group from cycle 4–5, which continued up to the point to equate that EtOH consumption by animals always housed in SE conditions (SE-SE group). Consistent with this finding, additional Bonferroni tests comparing SE-SE vs. EE-SE showed statistical group differences on cycle 4, but not on cycle 8, while significant differences in EE-EE vs. EE-SE EtOH intake emerged at the 8th cycle. Taken together, these results strongly suggest that losing EE during adulthood represents a risk factor triggering high binge-like EtOH consumption in mice pre-exposed to long-term iDID. Moreover, they indicate that the protective effect of EE exposure during adolescence and early adulthood on EtOH binge-like consumption might not be permanent. The present data are consistent with and support previous studies pointing to the negative impact of losing EE on drug intake. Switching mice from EE to SE conditions caused an increase in cocaine rewarding properties (Nader et al., 2012), a marked depressive-like phenotype characterized by low mobility in the forced swim test (Smith et al., 2017), and increased locomotor responses to amphetamine and saline when compared with chronically EE housed animals (Garcia et al., 2017).

EE exposure involves changes to a variety of neurobiological systems (Stairs and Bardo, 2009). To date, the underlying neurobehavioral mechanisms by which EE exerts both a protective and a therapeutic effect on drug and EtOH intake remain unclear, but long-term plastic adaptations in the reward and the stress neurobiological systems have been proposed. Thus, the impact of EE on heroin- and cocaine-seeking behavior and relapse might involve changes in mesocorticolimbic dopaminergic circuits (Solinas et al., 2008; Chauvet et al., 2011; Galaj et al., 2016), even when EE is introduced during adulthood (Del Arco et al., 2007; Segovia et al., 2010), and there is ample evidence indicating a primary anxiolytic role for EE (Benaroya-Milshtein et al., 2004; Peña et al., 2006, 2009; Sztainberg et al., 2010; Ragu Varman and Rajan, 2015; Bahi, 2017b). With the present results, we cannot conclude the precise neurobehavioral mechanisms underlying the observed therapeutic and protective impact of EE on binge-like EtOH intake; however, we discuss next EtOH consumption data associated with EE vs. SE conditions under the light of the observed EE impact upon anxiety, compulsive-like behaviors and novelty-seeking behaviors.

Regarding compulsivity-like behaviors and novelty-seeking data in our study, the HB test showed that adult animals (PND140) chronically housed in SE conditions (SE-SE group) exhibited a higher total number of head-dipping responses and higher repeated head-dipping than did animals in EE-EE housing conditions, which supports increased novelty-seeking and compulsivity-like responses, respectively, in SE-housed mice. Furthermore, animals reared in SE housing conditions during adolescence and then switched to EE conditions during adulthood (SE-EE group) showed a lower number of total head-dipping responses and head-dipping repetitions than their SE-SE counterparts, suggesting that access to EE ameliorated novelty-seeking and compulsive-like behaviors regardless of previous housing conditions. However, EE removal in the EE-SE group was associated with a significant increase in total head-dipping responses and number of repeated head-dips, which supports the hypothesis that loss of EE might have enhanced novelty-seeking behavior and compulsivity-like responses, respectively. Taken together, present data are consistent, and complement previous studies reporting positive EE effects on repetitive compulsive-like behavior (Bechard and Lewis, 2016; Bechard et al., 2016). Thus, EE-housed rats switched to isolated conditions increased novelty-seeking responses in the inescapable novelty test (IEN; Garcia et al., 2017), switching rats from isolated to enriched conditions decreased novelty-seeking responses (Garcia et al., 2017), and housing rats in enriched conditions from weaning to adulthood reduced novelty-seeking behavior in the OF test and locomotor activity cages (de Carvalho et al., 2010). Strong evidence supports head dipping in HB as a valid novelty-seeking trait measure (Vaglenova et al., 2004; Abreu-Villaça et al., 2006; Kliethermes and Crabbe, 2006; Mateos-García et al., 2015), however, some authors have suggested that the number of head dips might be indicative of a neophobic, rather than neophilic, response (Brown and Nemes, 2008). Keeping in mind those ideas, and considering this study did not provide a complete counter-balanced design to conduct EPM and HB tests, any conclusion derived from present HB data needs to be taken with caution.

We report here that adult animals (PND135) housed in EE conditions and drinking EtOH for a prolonged iDID procedure at the time of testing, showed fewer anxiety-like responses than did SE housed mice, indicated by a higher percentage of entries to open arms in the EPM. Furthermore, since animals reared in SE conditions and then housed in EE conditions during adulthood (SE-EE group) showed similar anxiety levels than EE-EE mice, we conclude that EE access might have anxiolytic-like effects regardless of specific housing conditions during adolescence. In contrast, loss of EE conditions during adulthood (EE-SE group) was associated with elevated anxiety-like responses compared to animals kept in EE conditions (EE-EE group). Importantly, group differences in anxiety were unrelated or secondary to altered locomotor activity, since total arm entries in the EPM were similar in all the groups. Moreover, we rule out a primary Et-OH-induced anxiolytic effect as the main factor explaining group differences since EE-EE animals drinking the lower EtOH amount showed the most reduced anxiety-like responses.

Present data are in agreement with previous research where the anxiolytic effect of EE, as measured by EPM, has been well documented (Benaroya-Milshtein et al., 2004; Peña et al., 2006, 2009; Sztainberg et al., 2010; Ragu Varman and Rajan, 2015; Bahi, 2017b), with slight differences due to strains used (Benaroya-Milshtein et al., 2004). Thus, in EE-reared mice it has been reported a reduction in stress induced by acute morphine treatment (Xu et al., 2014) and a reduction in abstinence signs induced by stress as well (Solinas et al., 2008; Chauvet et al., 2009), together with decreased hypothalamic-pituitary-adrenal (HPA) axis responses (Xu et al., 2007, 2014) and altered gene expression in the amygdala and the hypothalamus (El Rawas et al., 2011), all key brain regions involved in the stress response (Koob and Volkow, 2010).

Drug and EtOH addiction have been conceptualized as chronic disorders that involve elements of impulsivity and compulsivity, yielding a composite addiction cycle that develops over progressive stages, starting with a binge-intoxication phase leading to the pathological addiction state governed by enhanced compulsivity and increased sensitivity in the CRF-related brain anxiety systems (Koob and Volkow, 2010). Given, first, experimental evidence that loss of EE increases CRF mRNA expression in the Bed nucleus of the stria terminals (BNST; Nader et al., 2012); second, the role of the CRF system in the negative consequences of EE loss related to cocaine addiction (Nader et al., 2012); and third, available evidence demonstrating the key role of CRF in EtOH binge-like DID (Lowery-Gionta et al., 2012; Thiele and Navarro, 2014), there is a working hypothesis that the observed modulation of EtOH binge-like intake by EE conditions might be secondary to EE-elicited reductions in anxiety. Moreover, although speculative, under this hypothesis, EE exposure, during adolescence or during adulthood, might modulate activity in anxiety-related brain CRF pathways known to be early recruited during the EtOH addiction cycle (Lowery-Gionta et al., 2012; Thiele and Navarro, 2014), consequently reducing EtOH consumption and protecting vulnerable organisms from transition to addiction.

In summary, present data support the protective and therapeutic modulatory role of EE on binge-like EtOH consumption in iDID procedures, which successfully model human binge-like consumption. Early access to EE housing conditions during adolescence was associated with low EtOH binge-like intake accompanied by reduced anxiety-like, repetitive, compulsive-like and novelty-seeking behaviors. Interestingly, the modulatory role of early EE on EtOH binge-like consumption was not permanent since switching to SE conditions during adulthood blunted this effect. Furthermore, access to novel EE during adulthood ameliorated EtOH binge-like consumption in SE-reared mice, supporting a beneficial therapeutic role for EE exposure. In this study, we also showed that losing EE elicits increased EtOH consumption, anxiety, compulsivity, and novelty-seeking responses, while the opposite pattern was associated with EE access during adulthood. We propose the working hypothesis holding the primary impact of EE on anxiety-related, CRF brain systems known to modulate EtOH binge-like drinking during early, pre-dependent stages of the addiction cycle. Future research evaluating the neurochemical systems involved in the modulatory role of EE in EtOH binge-like drinking, anxiety, and/or compulsivity will provide key information for a deeper understanding of the role of EE in positively modulating early and transitional stages of the EtOH addiction cycle.

## Author Contributions

IC provided the overall coordination and supervision for the study. IC and ER-O were responsible for the study concept and design. ER-O and EA conducted behavioral characterization. LF was responsible for statistical analyses. IC and ER-O wrote the manuscript. All authors critically reviewed the content and approved the final version for publication.

## Conflict of Interest Statement

The authors declare that the research was conducted in the absence of any commercial or financial relationships that could be construed as a potential conflict of interest.
